# Comparison of single-dose radial extracorporeal shock wave and local corticosteroid injection for treatment of carpal tunnel syndrome including mid-term efficacy: a prospective randomized controlled trial

**DOI:** 10.1186/s12891-018-1948-3

**Published:** 2018-01-25

**Authors:** Pichitchai Atthakomol, Worapaka Manosroi, Areerak Phanphaisarn, Sureeporn Phrompaet, Sawan Iammatavee, Siam Tongprasert

**Affiliations:** 1 Department of Orthopaedics, Faculty of Medicine, Chiang Mai University, Chiang Mai, Thailand; 2 Division of Endocrinology, Department of Internal Medicine, Bangkok Chiang Mai Hospital, Chiang Mai, Thailand; 3 Department of Orthopaedics, Nakornping Hospital, Chiang Mai, Thailand; 4 Department of Rehabilitation Medicine, Faculty of Medicine, Chiang Mai University, Chiang Mai, Thailand

**Keywords:** Extracorporeal shock wave, Steroid injection, Carpal tunnel syndrome, Randomized controlled trial, Treatment

## Abstract

**Background:**

Recent studies have reported that radial extracorporeal shock wave therapy (rESWT) reduces pain and improves function in patients with mild to moderately severe carpal tunnel syndrome (CTS) compared to a placebo. However, most of those studies used multi-session rESWT combined with wrist support and evaluation of efficacy was limited to a maximum of 14 weeks.

**Methods:**

The prospective randomized controlled trial compared efficacy in relieving pain and improving clinical function between single-dose rESWT and local corticosteroid injection (LCsI) over the mid-term (24 weeks). Twenty-five patients with mild to moderately severe CTS were randomized to receive either single-dose rESWT (*n* = 13) or LCsI (*n* = 12). Primary outcomes were evaluated using the Boston self-assessment questionnaire (BQ), while secondary outcomes used the Visual analogue scale (VAS) and electrodiagnostic parameters. Evaluations at baseline and at 1, 4, 12 and 24 weeks after treatment were performed.

**Results:**

There was significantly greater improvement in symptom severity scores, functional scores and Boston questionnaire scores at weeks 12 to 24 in the rESWT group compared to the LCsI group. When compared to the baseline, there was significant reduction of VAS and functional score in the rESWT group at weeks 12 and 24. The LCsI group had no statistically significant differences in VAS reduction and functional score of the same period.

**Conclusions:**

Treatment of CTS using single-dose rESWT has a carry-over effect lasting up to 24 weeks suggesting that single-dose rESWT is appropriate for treatment of mild to moderate CTS and provides longer-lasting benefits than LCsI.

**Trial registration:**

(TCTR20150709001). Registered 9 July 2015

## Background

Carpal tunnel syndrome (CTS) is the most common entrapment neuropathy in the upper extremities [1]. Previous studies using varying based diagnostic criteria have reported population prevalence estimates from 2.7 to 14.4%, with a higher incidence in females than males among the elderly [2]. Specific causes of CTS are currently unknown, although evidence suggests that space occupying lesions can disturb blood circulation leading to demyelination of the median nerve and axonal loss [3–6]. Many risk factors are potentially associated with CTS including repetitive wrist motion, diabetes mellitus, hypothyroidism, rheumatism, obesity, arthritis, menopause and pregnancy [7–12].

Among the variety of optional treatments for CTS, local corticosteroid injection (LCsI) is widely used in mild to moderate cases and achieve improved symptom scores within 1 week [13]. A 2007 review by Cochrane reported greater symptom improvement 1 month after injection compared to placebo and significantly improved clinical outcomes compared to oral corticosteroids for up to 3 months [14]. Other publications have shown that LCsI provides benefits in terms of pain reduction and functional scores in patients with CTS. Those effects, however, were only reported to have occurred in the short term (up to 10 weeks) [15–17]. American Academy of Orthopaedic surgeons (AAOS) guidelines in 2008 and 2016 recommended LCsI or splinting for the treatment of CTS prior to considering a surgical option [18, 19].

There has been recent interest in extracorporeal shockwave therapy (ESWT) for CTS. Seok et al. demonstrated significant improvement in pain and symptom severity scores in CTS patients using focused ESWT (fESWT). At 3 months, however, symptom relief was not different from LCsI [20]. Several later studies have focused on the effect of radial ESWT (rESWT). CTS patients receiving 3 sessions of rESWT showed better clinical symptom improvement for 12–14 weeks compared to patients receiving sham ESWT [21, 22].

Patient compliance can affect the success of treatment [23, 24], so to reduce the period of time that a patient needs to repeat rESWT, we developed a single-dose rESWT for CTS and compared the efficacy of that treatment with LCsI up to the mid-term mark (week 24).

## Methods

### Design

This prospective, randomized, single-blind study was conducted at a tertiary level hospital. After receiving approval from our institutional review board, the Ethics Committee and registration at www.clinicaltrials.in.th (TCTR20150709001), we enrolled all new CTS patients who came to that hospital between January and June 2016 who gave their informed consent to participate in this study.

### Participants

Only CTS patients older than 18 years were included. CTS diagnosis was based on guidelines of the American Academy of Neurology (AAN) for CTS. Diagnostic criteria consisted of standard symptoms, provocative factors, mitigating factors and standard physical examination results [25]. Practice parameter for electrodiagnostic studies in CTS of the American Association of Electrodiagnostic Medicine 2002 was used as the criteria for neurophysiologic diagnoses [26, 27]. We included only patients with mild or moderate severe CTS following the electrodiagnostic criteria as specified by Sucher [27]. Patients with underlying metabolic disorders such as diabetes mellitus, genetic disorders, upper limb surgery, peripheral polyneuropathy, traumatic nerve injury, blood coagulation disorder while using anticoagulants, pregnancy, thrombosis, cancer or previous surgical treatment for cancer, treatment with ultrasound, cryo-ultrasound, oral steroids or Nonsteroidal anti-inflammatory drugs (NSAIDs) within 7 days prior to enrollment or local injection of corticosteroid for CTS in the previous year were excluded. In patients with bilateral CTS, the hand with the more severe condition was selected for evaluation of treatment outcome.

#### Randomization and allocation

Patients who met the inclusion and exclusion criteria were randomly assigned to either the rESWT or the LCsI group in blocks of four (number of subjects per block = 4, number of block = 7) using a random number generator (free program from www.randomization.com). The notes in each treatment was prepared and put into envelopes according to the allocation orders.

The nurse assistant who did not take the responsibility in the allocation method would open the envelope and informed PA or SP to do the treatment.

In data collection process, research assistants who were blinded to the randomization and treatment methods evaluated VAS and the Thai version of BQ at baseline (before treatment) and at weeks 1, 4, 12 and 24 following treatment for both treatment methods [28]. Electrodiagnostic evaluation was performed at baseline (before treatment) and 12 weeks after treatment in both groups.

Patients whose CTS symptoms progressed to the point they could not be tolerated and those who asked for additional or alternative treatment were eliminated from this study.

### Interventions

#### rESWT

Each patient in the rESWT group received shockwaves of continuous frequency and intensity (4 Bar, 15 Hz frequency, 5000 shocks, BTL-6000 SWT, radial shockwave mode). The probe was oriented perpendicular to the patient’s palm between the distal wrist crease and Kaplan’s cardinal line; ultrasound gel was used as a coupling agent. The duration of treatment was 3–7 min. A cold pack was applied for 15 min after rESWT.

#### LCsI

We used 1 ml. of triamcinolone (acetonide) 10 mg mixed with 1 ml of 1% lidocaine in a 3 ml disposable syringe. The injection was performed using a 25 gauge needle applied 1 cm proximal to the wrist flexion crease between the palmaris longus and flexor carpi radialis tendons. The angle of the needle was about 45 degrees distally and was advanced 1 cm, where it penetrated the flexor retinaculum. If paresthesia was provoked, we withdrew the needle and reinserted it 1 cm medial to the previous injection site. We advanced 1 cm at a time until the solution was injected. After injection, we instructed the patient to use their hand freely without splinting. Each patient was injected only once [29].

### Outcome measures

#### Primary outcome

BQ [30], the most commonly used instrument to assess improvement of clinical symptoms and functional recovery of patients with CTS, consisted of 11 questions covering symptom severity and 8 questions to evaluate functional status (functional score) which rate the level of difficulty to perform activities in daily life. The rating scale ranged from 1 to 5, with 5 being the most difficult. In our study, we used the Thai version of the BQ [28].

#### Secondary outcomes

1) VAS was used to evaluate the intensity of pain at rest on a 10 cm VAS in cm. The patient marked the scale on which the start point represented no pain and the endpoint represented maximum or intolerable pain. 2) Electrodiagnostic evaluation was performed by a Rehabilitation Medicine board qualified senior staff member. Median peak sensory latency in millisecond (ms), distal motor latency in millisecond (ms), sensory nerve action potential (SNAP) amplitude in microvolt (uV) and compound muscle action potential (CMAP) amplitude in millivolt (mV) were the parameters used in this study.

### Data analysis

Data were analyzed using Stata program (StataCorp. 2017. Stata Statistical Software: Release 15. College Station, TX: StataCorp LLC). For categorical variables, frequencies and percentages of were recorded; for continuous variables, means and standard deviations. Demographic data were analyzed using Fisher’s exact test for categorical variables, the independent t-test for normally distributed continuous variables and the Mann-Whitney U test for non-normally distributed continuous variables. The paired t-test was used to evaluate the outcomes at each follow-up visit (weeks 1, 4, 12 and 24 after treatment) compared to the baseline. Differences between the two treatment groups at each follow-up visit were analyzed using mixed-model analysis of repeated measures including measurement of the effect of treatment, time, severity and interaction between treatment and time. Statistical significance was accepted at *p* < 0.05.

Estimated sample size for two-sample comparison of means with repeated measures. The preliminary data of BQ scores were used to calculate the sample size in each group [alpha = 0.05 (two-sided), power = 0.9. mean in population 1 = 22.33, mean in population 2 = 29, SD in population 1 = 2.51, SD in population 2 = 3]. Estimated required sample size in each group was 4.

## Results

**Fig. 1:**
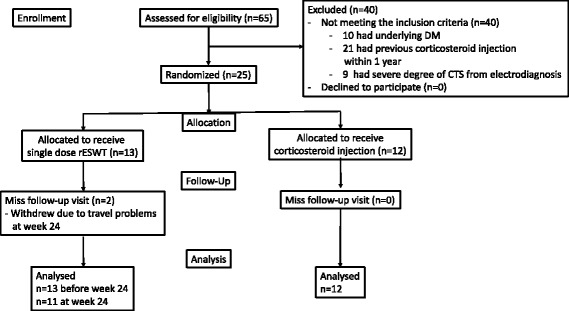
The participant enrollment flow diagram

After screening for eligibility, a total of 25 patients were enrolled in the study and randomly assigned to receive one intervention (rESWT or LCsI). During the follow-up period, 3 patients rejected to provide electrodiagnostic measurement at week 12 after the treatment. At the final follow-up period (week 24), another 2 patients withdrew due to travel problems and additional 2 patients in the LCsI group developed severe symptom progression which required additional treatment (Fig. 1).

There were no statistically significant differences between the groups in terms of demographic characteristics (age, gender, body mass index) or in clinical characteristics (lesion site, baseline of pain, symptom and functional score and severity determined by electrodiagnostic measurement (Table 1).

**Table 1:** Demographic and baseline characteristics of the groups

Characteristic	rESWT^a^ (*n* = 13)	LCsI^b^ (*n* = 12)	*p* value
Age, median (mean +/− SD), y	46 +/− 9	53 +/− 12	0.12
Gender, n (%)			0.16
Male	5 (62)	1 (8)	
Female	8 (38)	11 (92)	
Body mass index, (mean +/− SD)	28 (5)	24 (3)	0.09
Lesion site, n (%)			> 0.99
Right	9 (69)	8 (67)	
Left	4 (31)	4 (33)	
Unilateral	9 (69)	12 (100)	
Bilateral	4 (31)	0	
Severity, n (%)			> 0.99
Mild	5 (62)	4 (33)	
Moderate	8 (38)	8 (67)	
Baseline visual analogue scale, (mean +/− SD)	2.4 +/− 2.5	2.6 +/− 2.0	0.60
Baseline symptom severity score, (mean +/− SD)	21 +/− 6.4	22 +/− 5.1	0.63
Baseline functional score, (mean +/− SD)	14 +/− 3.2	12 +/− 4.1	0.16
Baseline Boston questionnaire score, (mean +/− SD)	35 +/− 8.5	34 +/− 8.5	0.77
Baseline peak sensory distal latency, (mean +/− SD)	4.5 +/− 0.72	3.7 +/− 1.3	0.057
Baseline SNAP^c^ amplitude, (mean +/− SD)	17 +/− 6.9	18 +/− 10	0.72
Baseline motor distal latency, (mean +/− SD)	4.5 +/− 0.35	4.7 +/− 0.90	0.57
Baseline CMAP^d^ amplitude, (mean +/− SD)	6.9 +/− 1.5	6.0 +/− 0.61	0.16

^a^rESWT = Radial extracorporeal shockwave therapy

^b^LCsI = Local corticosteroid injection

^c^SNAP = Sensory nerve action potential

^d^CMAP = Compound muscle action potential

There was a significant reduction of VAS and functional scores in the rESWT group at weeks 12 and 24 compared to baseline, while there was no significant change for the LCsI group. There were also significant reductions in symptom severity score and Boston questionnaire score at weeks 4, 12 and 24 in the rESWT group compared to baseline. In the LCsI group, there was significant reduction in terms of symptom severity score at weeks 1 and 4 as well as in the Boston questionnaire score at week 1 compared to the baseline. As to electrodiagnostic parameters, the rESWT group showed significant reduction in peak sensory distal latency at week 12 compared to the baseline as did the LCsI group. There were no significant changes from baseline in the other electrodiagnostic parameters in either group at week 12 (Table 2).

**Table 2:** Comparison of pre-treatment and post-treatment values of outcome variables in each group

		rESWT^a^ (*n* = 13)		LCsI^b^ (*n* = 12)	
Outcome variable	Follow-up sessions	Mean +/− SD	*p* value	Mean +/− SD	*p* value
Visual analogue scale	Baseline	2.4 +/− 2.5		2.6 +/− 2.0	
	Week 1	1.3 +/− 2.0	0.18	1.6 +/− 1.7	0.08
	Week 4	1.3 +/− 1.9	0.15	1.4 +/− 1.5	0.08
	Week 12	0.65 +/− 1.2	0.022^*^	1.9 +/− 2.7	0.52
	Week 24	0.35 +/− 0.81^c^	0.0075^*^	1.7 +/− 2.1	0.19
Symptom severity score	Baseline	21 +/− 6.4		22 +/− 5.1	
	Week 1	19 +/− 7.4	0.33	17 +/− 4.5	0.0047^*^
	Week 4	17 +/− 4.3	0.031^*^	17 +/− 5.1	0.011^*^
	Week 12	15 +/− 4.5	0.0082^*^	18 +/− 5.5	0.13
	Week 24	13 +/− 2.9^c^	0.0059^*^	19 +/− 7.9	0.20
Functional score	Baseline	14 +/− 3.2		12 +/− 4.1	
	Week 1	13 +/− 4.2	0.27	11 +/− 3.2	0.31
	Week 4	13 +/− 3.5	0.12	11 +/− 3.3	0.39
	Week 12	11 +/− 3.0	0.0065^*^	10 +/− 3.4	0.19
	Week 24	11 +/− 2.2^c^	0.0073^*^	13 +/− 7.0	0.65
Boston questionnaire score	Baseline	35 +/− 8.5		34 +/− 8.5	
	Week 1	32 +/− 10	0.28	28 +/− 7.0	0.037^*^
	Week 4	30 +/− 7.1	0.032^*^	28 +/− 8.0	0.05
	Week 12	26 +/− 6.8	0.0040^*^	29 +/− 8.2	0.13
	Week 24	24 +/− 4.7^c^	0.0037^*^	32 +/− 14	0.47
Peak sensory distal latency	Baseline	4.5 +/− 0.72		3.7 +/− 1.3	
	Week 12	4.1 +/− 0.74^d^	0.0047^*^	3.6 +/− 1.3	0.026^*^
SNAP^e^ amplitude	Baseline	17 +/− 6.9		18 +/− 11	
	Week 12	18 +/− 6.5^d^	0.66	21 +/− 12	0.28
Motor distal latency	Baseline	4.5 +/− 0.35		4.7 +/− 0.90	
	Week 12	4.2 +/− 0.42^d^	0.084	4.4 +/− 0.75	0.06
CMAP^f^ amplitude	Baseline	6.9 +/− 1.5		6.0 +/− 0.61	
	Week 12	6.9 +/− 1.6^d^	0.91	6.5 +/− 1.2	0.20

^*^p < 0.05

^a^rESWT = Radial extracorporeal shockwave therapy

^b^LCsI = Local corticosteroid injection

^c^*n* = 11

^d^*n* = 10

^e^SNAP = Sensory nerve action potential

^f^CMAP = Compound muscle action potential

A comparison of post-treatment values of outcome variables between groups at each follow-up period is provided in Table 3. There was a significant declination in symptom severity score, functional score and Boston questionnaire score between week 12 and week 24 in the rESWT group compared to the LCsI group. While the VAS did not show the statistically significant difference between two groups in this period of time. There was a greater reduction in peak sensory distal latency between baseline and week 12 in the rESWT group than the LCsI group. Mixed-model analysis of repeated measures found that severity from electrodiagnostic measurement (mild or moderate) did not affect all outcome parameters (*p* > 0.05) with the exception of SNAP amplitude (*p* < 0.05).

**Table 3:** Comparison of post-treatment values of outcome variables between both groups in each follow-up peroid

Outcome variable	Follow-up sessions	Coefficeint (rESWT^a^ VS LCsI^b^)	*p* value	95% CI
				ᅟ
Visual analogue scale	Baseline to week 1	−0.10	0.90	−1.7 to 1.5
	Week 1 to week 4	0 .049	0.95	− 1.6 to 1.7
	Week 4 to week 12	−1.0	0.23	−2.7 to 0.63
	Week 12 to week 24	−1.2	0.17	− 2.9 to 0.49
Symptom severity score	Baseline to week 1	2.6	0.27	−2.0 to 7.2
	Week 1 to week 4	1.5	0.53	−3.2 to 6.1
	Week 4 to week 12	−1.9	0.43	−6.5 to 2.8
	Week 12 to week 24	−5.1	0.036^*^	−9.8 to −0.33
Functional score	Baseline to week 1	0.43	0.81	−3.1 to 3.9
	Week 1 to week 4	−0.20	0.91	−3.7 to 3.3
	Week 4 to week 12	−1.8	0.32	−5.3 to 1.7
	Week 12 to week 24	−4.5	0.015^*^	−8.1 to −0.87
Boston questionnaire score	Baseline to week 1	3.0	0.43	−4.5 to 11
	Week 1 to week 4	1.3	0.74	−6.2 to 8.8
	Week 4 to week 12	−3.7	0.34	−11 to 3.8
	Week 12 to week 24	−9.5	0.015^*^	−17 to − 1.9
Peak sensory distal latency	Baseline to week 12	−0.24	0.022^*^	− 0.44 to − 0.034
SNAP^c^ amplitude	Baseline to week 12	−2.8	0.36	−8.9 to 3.2
Motor distal latency	Baseline to week 12	−0.038	0.84	−0.41 to 0.33
CMAP^d^ amplitude	Baseline to week 12	−0.44	0.34	−1.4 to 0.46

^*^p < 0.05

^a^rESWT = Radial extracorporeal shockwave therapy

^b^LCsI = Local corticosteroid injection

^c^SNAP = Sensory nerve action potential

^d^CMAP = Compound muscle action potential

## Discussion

Session rESWT is becoming a popular treatment for patients with mild to moderate CTS. Published results of prospective, randomized control trials have shown regression in pain intensity and improvement of clinical symptoms compared to sham rESWT. However, session rESWT requires patients to return to a medical center to receive treatments for 3 to 4 consecutive weeks [21, 22, 31], a situation that is reflected in reports of compliance affecting the success of treatment [23, 24]. We theorized that if a single-dose rESWT provides a good functional outcome in mild and moderate CTS patients, it might resolve the compliance problem by eliminating the need for repeated interventions. The levels and duration of efficacy of a single-dose rESWT treatment of CTS are still controversial, including rESWT efficacy compared to LCsI, a CTS treatment frequently recommended prior considering surgical options [18, 19], i.e., the relative efficacy of single-dose rESWT treatment compared to LCsI was still an open question.

To the best our knowledge, this study examines a new dimension in the treatment of CTS. Single-dose rESWT, which has a carry-over effect lasting up to 24 weeks, showed significant improvement in terms of clinical symptoms and functional recovery compared to LCsI from weeks 12 to week 24. Most previous studies have conducted the final follow up at week 12 or week 14 [20–22, 32, 33]. The methodology evaluated in this study showed the efficacy of single-dose rESWT in the absence of any other confounding treatment, e.g., the wrist splints used in combination with rESWT in other studies [21, 22, 31, 32, 34]. This study used mixed-model analysis of repeated measures to analyze differences between treatments which can detect the effect of time on the results of treatment while other cohort studies have used the Mann-Whitney U test [20, 21] and the independent t-test [31, 32].

One limitation of our study is the relatively low number of patients compared to other studies [20–22, 31–34], although our sample did have enough statistical power to detect the significant difference between groups at weeks 12 and 24. A second limitation is that the different dose intensity of rESWT might affect the results of treatment. Finally, long- term results, beyond 24 weeks, were not measured. Future studies should include larger numbers of patients, different treatment protocols and longer follow-up periods.

In our study, the pain relief benefit of rESWT appeared to begin at week 12 and to continue through week 24 compared to baseline, while LCsI initially provided statistically insignificant pain reduction which disappeared entirely by week 24. Compared to previous studies, the effect of pain reduction from session rESWT seemed to begin earlier in our study [21, 31, 32]. In terms of symptom severity and functional outcomes, with single-dose rESWT significant improvement compared to the baseline began at week 4, while the LCsI group had significant improvement at only week 1. Conversely, symptom and functional improvement were not significant after that time. This differs from Wo et al. which reported an earlier effect of session rESWT for symptom and functional improvement beginning at week 1 [21]. The differences in pain, symptom and functional improvement of single dose rESWT in our study compared to session rESWT in others studies might be the result of differences in the method of rESWT (single vs. session) as well as the effect of wrist splinting in the other studies (single-dose rESWT vs. session rESWT+wrist splinting) [21, 31, 32].

Our study found a significant decrease in peak sensory distal latency in both groups at week 12 compared to the baseline while other electrodiagnostic parameters showed no significant difference between the baseline and week 12. The only significant difference in improvement of electrodiagnostic parameters between the groups was the greater reduction in peak sensory distal latency in the rESWT group. Two other studies reported a significant increase in sensory nerve conduction velocity between baseline and week 12 with rESWT [21, 32]. One of those studies detected no difference between the baseline and week 12 for all electrodiagnostic parameters in a fESWT group [20], while the other study reported a significant decrease in CMAP and SNAP distal latency between baseline and week 12 in an ESWT group [31]. Taken together, these results seem to indicate that the effect of electrodiagnostic changes seems to be inconclusive. The reason the results of electrodiagnostic changes varied might be a reflection of the fact that the other studies also found no significant relationship between symptom severity score and electrodiagnostic findings [35, 36]. Additionally, one study reported that the electrodiagnosis could access only large myelinated nerves functions. Small non-myelinated sensory nerve functions, which are commonly associated with CTS symptoms, could not be evaluated by electrodiagnostic measurement [37].

Our study demonstrated that single rESWT provided greater benefits in term of symptom severity reduction and functional improvement compared to LCsI during weeks 12 and 24, while other studies have reported on maintenance of benefits from LCsI for only short periods (up to 3 months) [13, 14, 17].

The mechanisms by which rESWT helps CTS patients remain inconclusive. One experimental study reported that ESWT down-regulated the inflammatory effect by inducing tyrosine dephosphorylation of endothelial nitric oxide synthase which increases the level of nitric oxide. After that the amount of the nitric oxide suppresses NF-kappa B activation which inhibits LPS/IFN-gamma-elicited expression of genes in the inflammatory process [38]. A study in rat glioma cell line C6 also reported an anti-inflammatory effect caused by the same mechanism [39]. Previous reports have also described effects of ESWT including enhancing elimination of injured axons, promoting Schwann cell proliferation and increasing axonal regeneration [40–42].

The efficacy of ESWT relies on the dose intensity, duration and number of attempts. A recent study reported a longer-lasting effect with multiple-session attempts compared to a single-dose [22]. Our single-dose rESWT protocol had a higher dose intensity than that study (3–7 min 4 Bar, frequency: 15 Hz, number of shocks: 5000 shocks VS 4 Bar, frequency: 5 Hz, number of shocks: 2000 shocks) [22]. Our rESWT protocol provided a long-lasting effect, up to 24 weeks, without multiple-session attempts.

No serious complications in terms of severe pain or progression of symptoms occurred with single-dose rESWT. A few patients mentioned minimal pain during treatment, but that pain was relieved within a minute following application of a cold pack. To date, there have been no severe complications reported from using fESWT or rESWT to treat CTS patients [20–22, 31–34]. The literature does mention that transient pain, skin redness or small hematoma might present after ESWT, but those are typically resolved spontaneously [43].

## Conclusions

Based on the results of this study, single-dose rESWT provides longer-lasting benefits than LCsI in the treatment of mild to moderate CTS.

## Abbreviations

AAN: American Academy of Neurology
AAOS: American Academy of Orthopaedic surgeons
BQ: The Boston self-assessment questionnaire
CMAP: Compound muscle action potential
CTS: Carpal tunnel syndrome
LCsI: Local corticosteroid injection
NSAIDs: Nonsteroidal anti-inflammatory drugs
rESWT: Radial extracorporeal shock wave therapy
SNAP: Sensory nerve action potential
VAS: Visual analogue scale
